# Unintended pregnancy and subsequent use of modern contraceptive among slum and non-slum women in Nairobi, Kenya

**DOI:** 10.1186/1471-2393-14-224

**Published:** 2014-07-10

**Authors:** Jean Christophe Fotso, Chimaraoke Izugbara, Teresa Saliku, Rhoune Ochako

**Affiliations:** 1 International Consultant, Population & Reproductive Health, Nairobi, Kenya; and Concern Worldwide US, New York, USA; 2 African Population and Health Research Center, Nairobi, Kenya; 3 Twaweza East Africa, Nairobi, Kenya; 4 Population Services International, Nairobi, Kenya; 5 P.O. Box 34323, 00100 Nairobi, Kenya

**Keywords:** Unintended pregnancy, Unplanned pregnancy, Contraceptive use, Urban poor, Kenya

## Abstract

**Background:**

In spite of major gains in contraceptive prevalence over the last few decades, many women in most parts of the developing world who would like to delay or avoid pregnancy do not use any method of contraception. This paper seeks to: a) examine whether experiencing an unintended pregnancy is associated with future use of contraception controlling for a number factors including poverty at the household and community levels; and b) investigate the mechanisms through which experiencing an unintended pregnancy leads to uptake of contraception.

**Methods:**

Quantitative and qualitative data from a cross-sectional research project conducted in 2009/10 in two slum settlements and two non-slum settings of Nairobi, Kenya are used. The quantitative component of the project was based on a random sample of 1,259 women aged 15–49 years. Logistic regression models were used to assess the effect of unintended pregnancy on future contraceptive use. The qualitative component of the project successfully interviewed a total of 80 women randomly selected from survey participants who had reported having at least one unintended pregnancy.

**Results:**

Women whose last pregnancy was unintended were more likely to be using a modern method of contraception, compared to their peers whose last pregnancy was intended, especially among the wealthier group as shown in the interaction model. Among poor women, unintended pregnancy was not associated with subsequent use of contraception. The qualitative investigation with women who had an unplanned pregnancy reveals that experiencing an unintended pregnancy seems to have served as a “wake-up call”, resulting in greater attention to personal risks, including increased interest in pregnancy prevention. For some women, unintended pregnancy was a consequence of strong opposition by their partners to family planning, while others reported they started using contraceptives following their unintended pregnancy, but discontinued after experiencing side effects.

**Conclusion:**

This study provides quantitative and qualitative evidence that women who have had an unintended pregnancy are “*ready for change*”. Family planning programs may use the contacts with antenatal, delivery and post-delivery care system as an opportunity to identify women whose pregnancy is unplanned, and target them with information and services.

## Background

In spite of major gains in contraceptive prevalence over the last few decades, many women in most parts of the developing world and in sub-Saharan Africa in particular, who would like to delay or avoid pregnancy, do not use any method of contraception [1,2]. Each year, in the developing world, only about half of the women at risk of experiencing unintended pregnancy use a modern method of contraception [1]. As a result, women in developing countries have 75 million unintended pregnancies annually, representing about a third of all the pregnancies that occur in the region [3], a proportion which varies from as low as 10% in some sub-Saharan African countries to as high as 65% in others [4].

The health and social consequences of unintended pregnancy are amply documented, and its implications for the achievement of the Millennium Development Goals (MDGs) are now well understood [5,6]. Unintended pregnancy has been shown to adversely influence maternal and child health seeking behaviors, birth outcomes, and women’s quality of life [7,8], and to contribute to unsafe abortion [9]. Increasing the uptake of more effective contraceptive methods is thus a necessary first step towards reducing unplanned pregnancy and the vicious cycle of poverty and poor reproductive health [4,6].

Several studies have explored the association between contraceptive use and women’s risk for unintended pregnancy. These studies have generally related unintended conception among women to contraceptive behavior, types of contraceptives, as well as the consistency and correctness of use [2,4,8,10]. In contrast, much less is known about the possible effects of experiencing an unintended pregnancy on future contraceptive use, despite evidence that an unplanned pregnancy is a risk factor for a subsequent unplanned pregnancy [11]. Where evidence exists, it has largely drawn on data from western contexts [12,13], and focused primarily on unmarried adolescent girls, neglecting adult and married women who are also often at heightened risk for unintended pregnancy. In a study of 424 American women aged 14–25 years, Matteson et al. [12] concluded that a past unplanned pregnancy was not associated with contraceptive use. However, they recommended further studies on the relationship and speculated that factors that inhibit contraceptive use (e.g. lack of access, fear of side effects) may have overshadowed the motivation for contraceptive use provided by an unplanned pregnancy. In Orcutt & Cooper [13], American female adolescents who had experienced an unplanned pregnancy appeared to have the fastest increase in contraceptive uptake, but the authors suggested that the difference may be explained –at least partly- by the study design, in particular the time between the survey waves.

The current study attempts to extend understanding of the association of unintended pregnancy and contraceptive use by analyzing data collected from a sample of girls and women in Nairobi, Kenya. It hypothesizes that the occurrence of an unintended pregnancy is associated with future uptake of contraception, through mechanisms including a “wake-up call” for greater attention to personal risks and increased interest in pregnancy prevention. Its main objectives are thus to: a) examine whether experiencing an unintended pregnancy is associated with future use of contraception, controlling for a number factors including socio-economic status (SES) at the household and community levels; and b) investigate the mechanisms through which experiencing an unintended pregnancy leads to uptake of contraception.

### Context

Kenya offers a particularly interesting context for investigating women’s fertility practices and matters related to unintended pregnancy. The country has persistently boasted one of the highest contraceptive prevalence rates in Africa from 33% in 1993 to 46% in 2008/09, but the level of unintended childbearing has remained among the highest in the region and has declined only sluggishly during the 15-year period, from 50.9 to 42.6 percent [14,15]. Unintended fertility has contributed to Kenya’s rapid population growth and was cited as a major factor in the recent revision of Kenya’s projected population for 2050 from 54 to 83 million people [16]. Further, unintended pregnancy is a leading cause of unsafe abortion, contributing immensely to high levels of maternal mortality and morbidity in the country, with about 900 deaths occurring per 100,000 abortions in the country [17].

The high levels of unintended pregnancy in Kenya are related to poor access to, and use of, modern contraceptive services and products. On the supply side, facilities that provide subsidized family planning products and services regularly experience both stock outs and a dearth of qualified providers [18], while on the demand side, stigma, inadequate sexuality information, and cultural pressure inhibit the utilization of family planning services among women and girls in Kenya [19,20].

### Why should we pay attention to urban populations?

Sub-Saharan Africa is experiencing an urban explosion, with half of its population expected to be living in cities by 2030–2035. Following this trend, Kenya’s urban population grew from about half a million in 1960 to nearly 2.5 million in 1980, further increased to about 9 million in 2010 - constituting about 40% of the total population – and is projected to reach 40 million by 2050 [21]. The urban population explosion in most countries of sub-Saharan Africa has been occurring in the context of growing urban poverty, as manifested by the mushrooming of informal settlements and shantytowns that are characterized by poor access to healthcare and reproductive health (RH) services, early sexual debut and high-risk sexual behaviors [20,22]. Importantly, across sub-Saharan Africa, there are significant inequities in health and RH outcomes in urban settings, with the urban poor tending to have not only the lowest contraceptive use, but also the highest fertility and the highest unmet need for family planning [22,23].

## Methods

### Data

The current paper uses a mix of quantitative and qualitative data from a cross-sectional research project conducted in 2009/10 in two non-slum settings (Harambee and Jericho) and two slum settlements (Korogocho and Viwandani) in Nairobi, Kenya. The settlements form the Nairobi Urban Health and Demographic Surveillance System (NUHDSS), a research platform of the African Population and Health Research Center (APHRC). Korogocho and Viwandani are densely populated settlements characterized by high unemployment and poverty levels, crime, poor sanitation and high prevalence of risky sexual behaviors and poor sexual and reproductive health outcomes, compared to Nairobi as a whole [20,24]. Jericho and Harambee, on the other hand, are middle-class settings, and enjoy better health and other indicators [25].

The quantitative component of the research project was based on a sample of randomly-selected women aged 15–49 years, using a two-stage sampling procedure. In the first stage, 1,000 households from the two slum settlements and 1,000 households from the two non-slum settings were drawn from the NUHDSS, while the second stage consisted of a random selection of one eligible woman (usual resident aged 15–49) in each of the sampled households [26]. The sample size was based on the practice by the demographic and health surveys (DHS), which typically assume that to obtain reasonable precision for most indicators, at least 800 completed interviews of women 15–49 are needed in each domain. Accounting for possible missing data and non-responses, the sample size was set to 1,000 per area. The questionnaire sought information on respondents’ social, economic, demographic, pregnancy and birth histories (including miscarriages and abortions, stillbirths, and neonatal deaths), the intendedness of all pregnancies mentioned by the respondent irrespective of their outcomes, current use of contraception and particular methods used. A total of 1,962 women were successfully interviewed, yielding a response rate of 98.1%. For the purpose of this study, we restrict the sample to women whose last births or last pregnancy termination occurred in the five years preceding the survey. Besides, women who did not respond to the question on whether they were taking action to avoid pregnancy, had never had a pregnancy, had never had sex, or were pregnant at the time of the survey, were excluded, resulting in a final sample of 1,259 women. This dataset is used to address the first objective of the paper.

The qualitative component of the project successfully interviewed a total of 80 women (40 from the slum settings and 40 from the non-slum settings) randomly selected from survey participants who had reported having at least one unintended. In-depth interview was used to elicit information on views and experiences of unintended pregnancy and contraceptive use, among other topics. These interviews are utilized to investigate the possible mechanisms through which experiencing an unintended pregnancy hinders or leads to uptake of contraception. The sample size of 80 women, though arbitrary, was motivated largely by a concern with analytical expediency, aimed to help us achieve maximum variation sampling, which ensures representation of diverse dimensions of the issue being explored. Interviews were conducted using an in-depth individual qualitative interviewing guide that was administered in Swahili by four trained and highly-experienced female qualitative interviewers. The training was conducted by expert qualitative researchers and introduced the fieldworkers to the aims of the study, familiarized them with the study guide, and exposed them to guided dialogue techniques and critical tips for qualitative interviewing. The fieldworkers were mostly university graduates or students and were employees of APHRC’s NUHDSS at the time of the study. Overall, the research adhered to the guidelines for qualitative research review (RATS) which can be found at http://www.biomedcentral.com/authors/rats.

### Variables

The dependent variable is women’s current use of a modern contraceptive method (defined as sterilization, intrauterine device, injections, implant, pills, male condom, female condom, emergency contraception, lactational amenorrhea, and spermicides). The main predictor of modern contraceptive use is the planning status of the last pregnancy (mistimed/unwanted or not). The independent variables include place of residence (slum or non-slum); household wealth computed from household possessions, amenities and dwelling characteristics using principal components analysis [27] and recoded as tertiles (Low, Middle, High); respondent’s education, marital status, parity, age and ethnicity; and the time since the last birth or last pregnancy termination. The household wealth variable was computed from the overall sample (slum and non-slum settings). Of interest is the comparison of the influence on contraceptive use of SES at the household level (household wealth) and neighborhood levels (slums and non-slums). Fertility preferences and other determinants of contraceptive use such as knowledge about family planning, access to health information and services, discussion between couples and cultural barriers, are not available in the data, as the research project’s primary focus was to understand the magnitude of, and disparities in unplanned pregnancy in the study communities.

### Data analysis

The analyses were carried out in three steps. First, prevalence of unintended pregnancy and modern contraceptive use were estimated, and chi-square tests used to examine differences across the independent variables presented above. Second, a multivariate logistic regression analysis was conducted to assess the net effect of pregnancy *intendedness* on future contraceptive use. Third, interactions between unintended pregnancy on the one hand, and household wealth and slum/non-slum residence on the other hand, allowed us to examine the extent to which the effect of unintended pregnancy and future contraceptive use varies by socio-economic status (SES). The transcribed in-depth interviews (IDIs) were analyzed using an inductive approach involving thematic assessment of the narratives. Where applicable, quotations from the study participants are used to epitomize emerging issues and themes. To protect the identity of respondents, we have used pseudonyms throughout the paper. The STATA software was used for the management and analysis of the quantitative data.

## Ethical considerations

The project received ethical approval from the Kenya Medical Research Institute, and written informed consent was obtained from study participants before the interviews. Women in need of information or services were referred to appropriate facilities and organizations according to the protocols in place in the NUHDSS.

## Results

### Sample characteristics

As shown in Table 1, 58% of women from the study sample were from the slum settings. The household wealth variable was constructed on the sample of all households and as a result, is not evenly distributed among women in the sample. Nearly 35% of women had secondary-level education, while close to one-fifth had tertiary level education, a proportion comparable to the figure drawn from the urban sample of the 2008/09 Kenya demographic and health survey (18%). Majority of respondents were currently married, had given birth to two or three children, or were aged 25–34 years. Table 2 shows the characteristics of participants in the qualitative interviews. Majority of them had none or primary education, were currently married, had two children or more, and were aged between 35 and 49.

**Table 1:** Sample Characteristics for the analysis of last pregnancies; Nairobi, Kenya

**Variables**	**%**	**N**
Study site		
Slum	57.7	727
Non-slum	42.3	532
Household wealth^1^		
Lowest (Poor)	37.3	470
Middle	31.9	401
Highest (Rich)	30.8	388
Education		
None/primary	46.0	579
Secondary	34.5	434
Tertiary	19.5	246
Parity		
‘0-1	31.5	396
2-3	48.8	614
4+	19.8	249
Age		
<25	18.6	234
25-34	43.0	541
35-49	38.4	484
Marital status		
Currently married	61.7	777
Formerly married	22.2	279
Never married	16.1	203
Ethnicity		
Kikuyu	33.8	425
Luya	18.0	227
Luo	16.9	213
Kamba	19.2	242
Others	12.1	152
Time since last birth/pregnancy termination		
<18 months	27.3	344
18-35 months	22.6	284
36-59 months	50.1	631
N		1,259

^1^The distribution is 35.6% (Lowest), 32.2% (Middle), and 32.2% (Highest) in the slum setting; and 39.7% (Lowest), 31.4% (Middle), and 28.9% (Highest) in the non-slum sites.

**Table 2:** Background characteristics of women involved in the qualitative investigation

**Variables**	**N**	**%**
Education		
None/primary	37	46.3
Secondary	28	35.0
Tertiary	15	18.8
Marital status		
Currently married	47	58.8
Formerly married	15	18.8
Never married	18	22.5
Parity		
‘0-1	22	27.5
2-3	31	38.8
4+	27	33.8
Age		
<25	20	25.0
25-34	26	32.5
35-49	34	42.5
Residence		
Slum settings	41	51.3
Non-slum settings	39	48.8
N	80	100.0

### Patterns of unintended pregnancy and modern contraceptive use

Table 3 shows the magnitude of unintended pregnancy in the study areas, and its bivariate association with background characteristics. About 24% of women reported that their last pregnancy was unintended (either mistimed or unwanted), with higher levels in non-slums compared to slum areas (p = 0.022). Noticeably, the results show a pattern of steady decline of unintended pregnancy with increased household wealth (p = 0.011). The level of unintended pregnancy was highest among never married individuals (61.6%).

**Table 3:** Patterns of unintended pregnancy and contraceptive use among women in slum and non-slum settings of Nairobi, Kenya

	**Unintended pregnancy**	**Current modern contraceptive use**
Overall	23.7	47.6
Study site	p = 0.022	p = 0.041
Slum	21.3	45.1
Non-slum	26.9	50.9
Household wealth	p = 0.011	p = 0.287
Lowest	27.2	50.0
Middle	24.2	47.6
Highest	18.6	44.6
Education	p = 0.864	p = 0.215
None/primary	23.0	44.9
Secondary	24.2	49.8
Tertiary	24.4	50.0
Parity	p = 0.000	p = 0.000
‘0-1	35.4	46.7
2-3	16.6	52.8
4+	22.5	36.1
Age	p = 0.000	p = 0.000
<25	37.0	52.6
25-34	21.4	56.0
35-49	19.2	35.7
Marital status	p = 0.000	p = 0.000
Currently married	13.1	54.7
Formerly married	25.5	33.0
Never married	61.6	40.4
Ethnicity	p = 0.000	p = 0.552
Kikuyu	20.2	46.6
Luya	30.0	46.7
Luo	33.8	48.8
Kamba	19.0	51.7
Others	17.1	43.4
Time since last birth/pregnancy termination		
<18 months		53.5
18-35 months		52.8
36-59 months		42.0
N	298	599

Table 3 also shows that modern contraceptive prevalence rate (CPR) stands at 47.6% in the study areas, with higher levels recorded in the non-slum settings (p = 0.041). Women’s parity, age and marital status display statistically significant associations with use of a modern method of contraception (p = 0.000 in all three cases), with the highest rate recorded among currently married women (54.7%). As expected, use of contraceptive decreases with the length of time since the last birth or last pregnancy termination (p = 0.000).

### Unintended pregnancy and contraceptive use

Table 4 presents the results of the multivariate analysis. The key finding is that controlling for possible confounding factors, women whose last pregnancy was either mistimed or unwanted, were more likely to be using a modern method of contraception, compared to their peers whose last pregnancy was wanted (p = 0.030).

**Table 4:** Multivariate results of the determinants of contraceptive use among women in slum and non-slum settings of Nairobi, Kenya

	**Main effects**		**Interaction: Unintended pregnancy and slum/non-slum residence**		**Interaction: Unintended pregnancy and household wealth**	
	**Odds ratio**	**P-value**	**Odds ratio**	**P-value**	**Odds ratio**	**P-value**
Unintended pregnancy [Ref: No]						
Yes	1.41	0.030*	1.53	0.034*	0.84	0.458
Study site [Ref: Slum]						
Non-slum	1.50	0.012*				
Household wealth [Ref: Low (Poor)]						
Middle	0.96	0.785				
High (Rich)	0.81	0.160				
Education [Ref: None/primary]						
Secondary	1.14	0.371				
Tertiary	1.08	0.716				
Household size [Ref: 1–2]						
3-5	1.24	0.323				
6+	1.60	0.047*				
Age [Ref: 35–49]	1.00					
<25	2.59	0.000***				
25-34	2.48	0.000***				
Marital status [Ref: Never married]	1.00					
Formerly married	1.06	0.237				
Currently married	2.71	0.000***				
Ethnicity [Ref: Kikuyu]						
Luya	0.85	0.344				
Luo	0.91	0.623				
Kamba	0.92	0.614				
Others	0.69	0.067^†^				
Time since last birth/end of pregnancy [Ref: <18 months]						
18-35 months	1.05	0.769				
36-59 months	0.89	0.463				
Interaction: Unintended pregnancy and slum/non-slum residence						
Unintended pregnancy × Non-slum			0.83	0.505		
Interaction: Unintended pregnancy and household wealth						
Unintended pregnancy × Middle					2.50	0.006**
Unintended pregnancy × Rich					2.22	0.023*

^†^p < .10; *p < .05; **p < .01; ***p < .001.

Table 4 also shows that non-slum residents are more likely to use modern contraceptive, compared with slum residents (p = 0.012). This effect of SES observed at the community level (non-slum being considered wealthier than slum residents) is not apparent at the household and individual levels. Indeed the effect of household wealth is not statistically insignificant (as noted in the bivariate analysis), while that of education shows no difference between secondary and tertiary education. The ethnic differences in the use of contraception or the effect of time since the last birth/pregnancy termination are not statistically significant in the multivariate analysis. Noticeably, marital status and age remain the strongest predictors of modern contraceptive use. Currently married women were about 2.7 times as likely as their formerly married counterparts to be using a modern contraceptive method (p = 0.000). As for age, women aged less than 35 years were nearly 2.5 times more likely than their older peers (aged 35–49), to be using a modern method of contraception (p = 0.000).

### Does the association vary by SES?

In Table 4, the interaction between unintended pregnancy and slum/non-slum residence was not statistically significant, indicating that the effect of unintended pregnancy does not vary significantly between the slum and non-slum areas. The interaction with household wealth on the other hand, shows that the effect of unintended pregnancy is significantly stronger among non-poor households (middle and rich households). It is notable that among women from poor households, experiencing an unintended pregnancy was not statistically associated with future use of a method of contraception.

### Unintended pregnancy and future contraceptive use practices: Possible mechanisms

Findings from the qualitative study suggest that several women who reported an unintended pregnancy did not want to have a repeat episode of the same. While evident in many of the narratives we collected, one of the most poignant articulations of this point was by a 25-year old respondent who noted ‘*I was confused and helpless …when I found that I was pregnant. It was not easy for me. I would not want to go through that again’.* The women we studied generally acknowledged being deeply troubled and distressed by their unintended pregnancies. Several of them admitted panicking, experiencing anxiety, and not knowing who to talk to or where to seek help. Largely because of shame and stigma, many of the women interviewed could not tell family members, partners and friends of their plight.

The narratives of the women we interviewed generally suggested that the experience of an unintended pregnancy jolted them and presented them with both a wake-up call and an opportunity to reassess their relationships, sexual and contraceptive practices and behaviors, and life situations. Driving this point home, a 28-year unmarried respondent noted: ‘*It was scary for me, and taught me a lot… about the boy I had called my friend. From that pregnancy, I learnt a lot about men that has stayed with me*’. There was also a 25-year old married respondent who reported that she made a decision to avoid unprotected sex following her unplanned pregnancy. Experiencing an unintended pregnancy also encouraged women to discuss family planning and contraception with their partners and to demand the use of condoms to avoid another pregnancy. As one 27-year old unmarried respondent put it: ‘*The pregnancy disturbed me and I am like I won’t want to go through that kind of experience again. My partner told me that I have changed, I have become rebellious, I don’t want to do some things and it is due to the fact that when my mind tells me I am not protected, that is it. And so I am like if you don’t want to use a condom or something, I don’t want it. Sometimes we end up fighting’.*

In explaining their decision to begin to use a contraceptive method, several women in the study often made reference to the crisis and difficulties they faced when they found out about their accidental pregnancies. For one married 37-year old mother of four, her unintended pregnancy caused her so much anxiety and worry. She unwillingly carried the pregnancy to term, but obtained tubal ligation immediately after the delivery. She noted: ‘*I fear [to get pregnant], so I had to go for tubal ligation so I cannot have other children now… I would have liked to have as many as possible children but when I look at the problems I would go through, I am better off now’*. The prevention of future unintended pregnancy and abortion was considered important. Another currently married 35-year-old respondent noted: ‘*Yes, I don’t want anything to do with that at all. Abortion is a risky affair and anything can happen to you; it is something you are doing but scared of the eventuality. The processes you pass before you are able to secure an abortion whether it is done by the doctor or not, are so long and the whole situation is really scary. It is something you are conscious about that you are doing some wrong*.’

Following an unintended pregnancy, some women also decided to use contraceptives because it protected their reputation. When asked what bothered them most about their unintended pregnancy, some of the women focused on issues of perception by the community, peers, and family. A good example is this 23 year old, who told us: ‘*When I found out that I was pregnant, my greatest worry was what people would think of me…that I was a prostitute. If they found out about the pregnancy, they would say that one is a spoilt (immoral) girl*’. Similar sentiments were expressed by a 40-year-old respondent that she accidentally became pregnant soon after her husband’s death, when the mourning was still ongoing.

Unwanted pregnancies also worsened livelihoods for some of the women. For instance, one 34-year-old respondent told us that she already had two children, when she became pregnant unintentionally. While she carried the third pregnancy to term, she told us: *‘I struggle every day to support them. I knew three children will be too much for me, that is why I did not… another child.’* As this respondent told us, she had no choice but to go for tubal ligation.

However, not all the women who experienced an unplanned pregnancy used modern method of contraception afterwards. While some respondents, particularly unmarried young women resorted to abstinence, others reported that they did not use contraceptives because it was dangerous and had potential to harm them. There were also women who reported that they started using contraceptives following their unintended pregnancies, but discontinued after experiencing side effects.

## Discussion

The focus of this study is on the extent to which unintended pregnancy affects future use of modern contraceptive methods. A major limitation, which also applies to most investigations on unwanted fertility, is the difficulty to accurately capture the very concept of unintended pregnancy, as a number of authors have reported a tendency of women to revise the status of a pregnancy after birth [28,29]. Further, due to the small sample size of this study, we combined mistimed and unwanted pregnancies, and considered their joint influence on contraceptive use. As acknowledged by D’Angelo et al. [30], mistimed pregnancies and unwanted pregnancies may have different consequences and may affect subsequent use of contraception differently.

Another limitation of the analysis is related to the possibility that a woman may change her decision to use or not to use a contraceptive method more than once during the period between the birth of her last child or the termination of her last pregnancy, and the time of the interview. We sought to address this last issue using a multivariate model that controls for the length of time since the last birth or last pregnancy termination. Also, there may be a bi-directional relationship between unintended pregnancy and contraceptive use, with unobserved factors affecting both unintended pregnancy and contraceptive use. Our qualitative investigation sought to shed light on the specific influence of unintended pregnancy on future contraceptive use. Finally, the potentially higher exposure of the study population to health information and services suggests that our findings may vary in a different context.

Despite these limitations, this study provides important insights. The frequency of unintended pregnancies recorded in the study sites (about 24%) is lower than the figure reported in the 2008/09 Kenya Demographic and Health Survey for urban Kenya and Nairobi (around 29%), while the contraceptive prevalence rate in the study settings (about 48%) is comparable to the value for urban Kenya (46.6%) and Nairobi (49%) from the 2008/09 KDHS [31]. The data also reveal that about 70% of all unintended pregnancies among women with parity three or lower were mistimed, pointing to unmet need for spacing, whereas nearly 55% of all unintended pregnancies among women with parity four or higher were unwanted, suggesting a higher level of unmet need for stopping child bearing (not shown in Table 3). While the study shows that unintended pregnancy decreases with wealth, a result also observed in urban Kenya [30], it also reveals a higher prevalence in non-slums areas than in slum settings as per the bivariate results, a finding contrary to expectation, given the evidence that the slums of Nairobi are hubs of deprivation and risky health behaviors [20]. Contraceptive use on the other hand, is higher in non-slum areas, but is not significantly related to household wealth.

Importantly, the study indicates that controlling for possible confounders, women whose previous pregnancy was unintended were more likely to be using a modern method of contraception at the time of the survey. This finding is contrary to the results reported by Matteson et al. [12] and Orcutt & Cooper [13]. The interactive models – designed to illuminate the differences by SES in the size and direction of the association - reveal that among the non-poor women (middle and high wealth tertiles), a past experience of an unintended pregnancy is associated with higher use of modern contraception, while among urban-poor women (lowest wealth tertile), unintended last pregnancy is not significantly related to use of modern contraception, a finding which is likely to be explained by poor access to family planning services among the urban poor and the economic shock created by the unintended pregnancy or birth [22]. This finding suggests that in the absence of an intervention to increase the uptake of contraception, poorer women may be trapped in a vicious circle of poverty and unintended child bearing.

The qualitative investigation with women who had an unplanned pregnancy confirms some of the theoretical explanations identified in the literature. Experiencing an unintended pregnancy seems to have served as a “wake-up call”, resulting in greater attention to personal risks, including increased interest in pregnancy prevention [32], making the unintended pregnancy a “lesson moment”, in the sense that it provided motivation to make better reproductive health decisions [12]. Women who experienced an unplanned pregnancy feared they would not be financially able or ready to meet the costs of another pregnancy/child; others were concerned by the fact they were still living with parents or guardians, or that it was shameful to bring up a child as a single parent largely due to stigma associated with out-of-wedlock childbearing. Other respondents felt empowered to discuss family planning with their partners. To a large extent, the feeling of ‘never again’ was a great motivation for some women to begin thinking contraceptive use in general, or more effective methods in particular. By contrast, when factors underlying poor contraceptive use prior to the unplanned pregnancy remain unaddressed, the anticipated increased likelihood of contraception use may not be recorded [13]. For other women however, the unintended pregnancy may be a consequence of strong opposition to family planning, whether due to health concerns or other reasons. In this instance experience of an unintended pregnancy might be associated with reduced subsequent use of contraception compared to women who experienced a wanted pregnancy.

## Conclusion

Kenya, as many other African countries, continues to experience high levels of unintended pregnancies, with predictable adverse consequences on fertility decline and population growth. This study provides quantitative and qualitative evidence that women who have had an unintended pregnancy are “*ready for change*”. Family planning programs may use the contacts with antenatal, delivery and post-delivery care system as an opportunity to identify women whose pregnancy is unplanned, and target them with information and services, thereby strengthening the integration of family planning with maternal and child health services [33]. There is also urgent need for concerted effort to address the barriers that women face in accessing quality sexual and reproductive health information and services.

## Abbreviations

APHRC: African Population and Health Research Center; CPR: Contraceptive Prevalence Rate; DHS: Demographic and Health Surveys; IDIs: In-Depth Interviews; KDHS: Kenya Demographic and Health Survey; MDGs: Millennium Development Goals; NUHDSS: Nairobi Urban Health and Demographic Surveillance System; RH: Reproductive Health; SES: Socio-Economic status.

## Competing interests

The authors declare that they have no competing interests.

## Authors’ contributions

JCF and CI conceived the study; JCF carried out the quantitative analysis; CI and TS conducted the qualitative analysis; JCF and CI wrote the manuscripts with inputs from TS and RO in terms of literature review; all authors reviewed and approved the final draft of the paper.

## Pre-publication history

The pre-publication history for this paper can be accessed here:

http://www.biomedcentral.com/1471-2393/14/224/prepub
